# An experimental investigation examining the usage of a hybrid nanofluid in an automobile radiator

**DOI:** 10.1038/s41598-024-78631-9

**Published:** 2024-11-11

**Authors:** Amr M. Hassaan

**Affiliations:** Higher Technological Institute (HTI), 10th of Ramadan City, Egypt

**Keywords:** Radiator, Coolant, Hybrid, Nanofluids, Convection, Heat transfer, Enhancement, Mechanical engineering, Nanoscale materials, Nanoparticles

## Abstract

Several modifications have been made to the radiator’s dimensions and materials as part of the evolution of the automotive cooling cycle. Coolant is an important factor that greatly affects the efficiency of the cooling cycle. In applications involving heat transmission, nanofluids have become a viable possibility coolant. Two distinct types of nanoparticles floating in the base fluid make up the hybrid nanofluid, a newly invented class of nanofluids. Tests of hybrid nanofluids as a working fluid substitute for conventional fluids have been assisted by the current study. In the radiator of a 2005 Honda, the MWCNT–Al_2_O_3_/water nanofluid was tested at various volumetric concentrations (Φ) using a 50:50 mixing ratio. The outcomes of the experiments were compared with those obtained by using pure water. The radiator’s performance was evaluated by adjusting the fluid flow rate and operating the fluid at two distinct temperatures (60, 80 °C). The outcomes demonstrated that the convection heat transfer coefficient increased with a ratio reached 28.5% over the distilled water at the same temperature and flow rate. Both effectiveness and the Nusselt number had improved, coming in at 22.54% and 23.74%, respectively. Depending on the fluid concentration there is an increase in the pressure drop up to 24% than ordinary fluid. It discovered considerable agreement between the research outcomes by comparing them with earlier publications. An experimental correlation was inferred from the results to estimate the Nusselt number as a function of the Reynolds number and (Φ).

## Introduction

One of the contemporary issues that has been preoccupying scientists for quite some time iss how to reduce energy consumption and raise the performance of energy-consuming systems to reach the optimum efficiency limit for these systems^1^. Thermal systems are considered one of the most important systems consuming energy^2,3^. Scientists are investigating how to improve the thermal performance of these systems, as this is one of the important sources of energy savings. One of the thermal systems under study for a period until now is improving the thermal performance of the vehicle cooling cycle^4^. The use of non-traditional cooling fluids is one of the means that helps increase the performance of the cycle and contributes to the energy conservation process. Nanofluids have many advantages in heat transfer processes, as their thermal conductivity is higher than old fluids^5–7^. A hybrid nanofluid is a fluid in which particles of two or more nano-sized materials are added and dispersed in a conventional fluid to improve the properties of this fluid^8^. A 20% CHT enhancement with a minimum friction factor of 12% was observed by Hussein et al.^9^ in their water-TiO_2_ experiment at 1–4% volume concentration. TiO_2_- H_2_O/EG nanofluid was used in Devireddy et al.^10^’s investigation of a car radiator, and the researchers found that it improved heat transmission by up to 37% compared to the base fluid at 0.5 vol%. In an experiment conducted by Selvam et al.^11^ using graphene (GNP) - Water/EG based nanofluid in a car radiator at various flowrate and inlet temperatures, it was discovered that a 0.5 vol% improvement in entrance temperature led to a 104% improvement in OCHT. When EG-H_2_O based GO nanofluids of 0.1% weight were used in an investigation by Prasanna et al.^12^ in a vehicle radiator system, the researchers found that the greatest improvement was 71.1% at lower flow rates and constant entrance temperatures. According to the results of the experiments, the average CHT increases directly as nanofluid volume percentage and Re increases, and it is found that the average CHT is improved to 196.3% for 0.5 vol%, compared to base fluid. M’hamed et al.^13^ performed an experimental analysis on the thermal behavior of H_2_O/EG based MWCNT nanofluid in a radiator for different liquid and air flow rates. Fe_2_O_3_-TiO_2_/water HyNf was evaluated in an aluminum tube radiator by Abbas et al.^14^ who found that the HTR and Nu increased by 26.7% & 20.03% in comparison to base fluid for a 0.009 vol% at 56 °C, 15 LPM. Additionally, it was shown that performance degrades above 0.009% volume concentration due to nanoparticle blockage, which lowers HyNf stability. ZnO and AlN NPs used in ethylene glycol base coolant fluid are studied by Bargal et al.^15^ in a 50:50 ratio. A range of 0.2–0.5% is designated as the nanoparticle volume concentration. The experimental results show that the RHT of the base fluid and nanofluids increases at high flow rates. By utilizing MWCNT nanofluid in the automobile radiator, Sivalingam et al.^16^ extend the scope of their research. The highest increase in friction factor was 0.25 for an input coolant temperature of 343 K and a concentration of MWCNT nanoparticles of 0.6%. Nusselt number addition is 50% more than with the traditional base fluid. The thermal behavior of ethylene glycol and distilled water, as well as the composition of EG/Distilled water-based aluminum sulfate and magnesium sulfate nanofluids and their produced hybrid nanofluid, are studied by Efemwenkiekie et al.^17^ in an automotive radiator. Arif et al.^18^ demonstrate that using three-fluid hybrid nanofluids based on water boosts RHT by up to 33.57%. By incorporating nanoparticles into the base fluid, Ajeeb et al.‘s experimental and computational results^19^ demonstrate that the compact plate heat exchanger’s thermal performance increases. According to the research by Li et al.^20^, silicon carbide and MWCNTs nanofluids had an overall convective heat transfer coefficient that was 26.5% greater than pure ethylene glycol under identical circumstances. In an experiment using a vehicle radiator’s laminar flow regime, Naraki et al.^21^ look at the overall CHT of copper oxide/water fluids. Using nanofluid increases the convective CHT by up to 8% as compared to the base fluid DW at a concentration of 0.4% nanofluid. Nanofluids utilized for cooling car engines are studied by Kumar et al. in their work on heat transfer analogies^22^. An important assessment of the role of nanofluids in automotive radiator cooling is made. Graphene and H_2_O + EG nanofluid are used in an experiment by Naveen et al.^23^ to study the convective heat transfer characteristics of an automotive radiator. At increased mass flow rates and volume concentrations of nanofluid, the Nusselt number increases by 54.40%. The radiator’s OCHT was examined by Akash et al.^24^ using three nanofluids: copper, aluminum, and MWCNT nanofluids. They discovered that MWCNT offers the highest boost of 40%, followed by copper with 29%, while aluminum nanofluid raises OCHT to roughly 25%. Ajeeb et al. conducted investigations^25,26^. They showed that pool boiling is one of the processes and systems that can benefit greatly from the recyclability of nanofluids due to their high yield. The pool boiling heat transfer performance of HNFs and Mono NFs was much better than that of this BF.

By looking at the literature contained in the research and others, hybrid nanofluids have a prominent role in the process of improving heat transfer by using them as operational fluids in automobile radiators. However, to the best of the author’s knowledge, the literature that has dealt with the use of hybrid nanofluids (MWCNT–Al_2_O_3_/water) is very limited. To evaluate the hybrid nanofluid (MWCNT–Al_2_O_3_/water), the author carried out experimental study and contrasted its performance with that of the conventional fluid that was being used (distilled water). A variety of nanofluid concentrations were employed, and the impact of altering the fluid flow rate and temperature on the heat transfer process was also examined.

## Experimental testing device

**Fig. 1:**
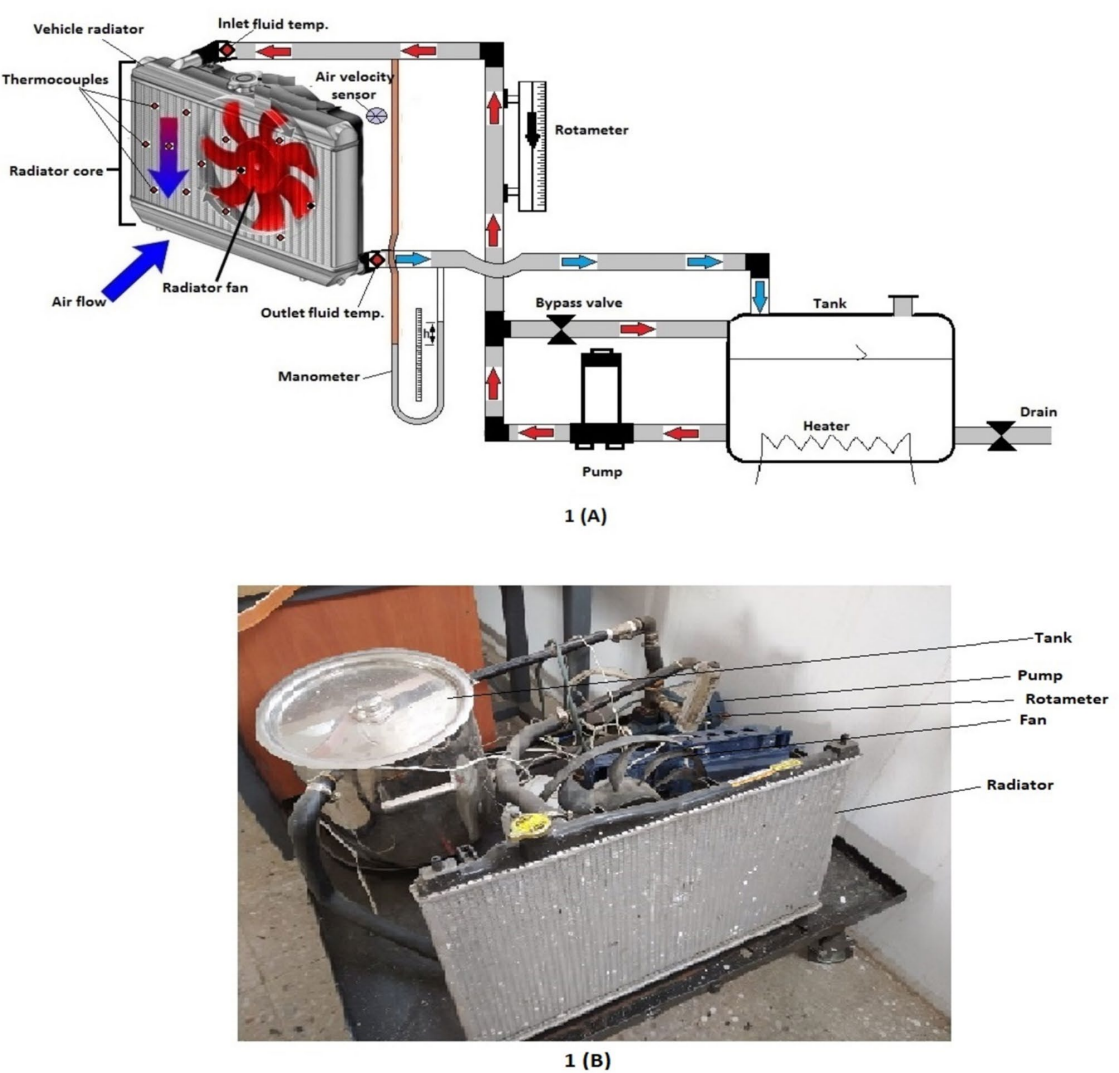
(**A**) Experimental test rig schematic diagram, (**B**) An image of the experimental apparatus.

To achieve the goal of this study, it was necessary to manufacture a cooling cycle that simulates the cooling cycles found in many vehicles. A test platform was equipped with several devices necessary to evaluate heat transfer performance. Instead of employing conventional fluids like distilled water or ethylene glycol to function as operational fluids inside the cycle, the study focuses on substituting hybrid nanofluids. (MWCNT–Al_2_O_3_/water) is the hybrid nanofluid that was utilized in these experiments. Hybrid nanofluids were manufactured at more than one volume concentration, and the thermal performance of the manufactured fluids was tested. One of the traditional fluids widely used in cooling cycles is distilled water, so it was necessary to test its thermal performance in the same manufactured cycle to compare its results in the heat transfer process with synthetic nanofluids. Figure 1 displays an image of the study’s equipment as well as a diagrammatic depiction of the experimental test apparatus. The created fluids are heated inside a tank made of stainless steel with a capacity of 10 l. The tank is equipped with a pair of electric heaters, each with a capacity of 1.5 kilowatts. Each heater is individually connected to a thermostat to control the temperature of the fluids flowing within the circuit. The tank is open at the top to charge fluids and also see the level of the fluid inside the tank. The tank is completely insulated from the outside with a thick layer of glass wool to maintain the temperature inside the tank. At the end of the tank, a valve was placed to empty the tank when needed. In all experiments conducted, the temperature of the fluids was set to 60 and 80 °C through a thermostat.

Fluids are circulated through the manufactured device utilizing a centrifugal pump with a capacity of 0.5 hp. One of the variables being studied in this research is the fluid flow rate and its effect on the heat transfer process. The fluid flow rate is changed through a valve placed on the fluid exit line from the pump. To preserve and protect the pump, a bypath was created to empty the fluid into the tank instead of loading it onto the pump. A rotameter is used to measure the flow rate of fluid in the cycle. The fin-flat tube radiator with louvered fins from a 2005 Honda Civic was used in the study. The radiator characteristics utilized in the configuration are displayed in Table 1. An air fan was placed in front of the radiator to study heat transfer by forced convection. Although the fan speed can be controlled at values up to 20 m/s, the experiments conducted were at the same air speed, which is 15 m/s. Air speed is measured by a hot wire anemometer. The temperature of the cooling fluid entering and leaving the radiator, as well as temperature fluctuation on the radiator surface, are measured using thermocouples of type k. At both the radiator’s entrance and output, the forced air temperature is recorded. A U-shaped manometer was connected to the radiator’s inlet and exit point to detect the pressure loss caused by the coolant flow inside the radiator.

**Table 1:** The radiator’s geometrical features.

Geometrical aspects	Value	Geometrical aspects	Value
Radiator		Fins	
Material.	Aluminum	number of Fins.	65
Core size, (mm^3^)	350 × 660 × 16	Width of fin, (mm)	7.5
Number of tubes.	64	Fin length, (mm).	320
cross-section shape of tube.	Rectangular	length of Fin, (mm).	16
cross-section size of tube, (mm^2^).	16 × 2.7	thickness of fin, (mm).	0.1
Length of tube, (mm).	350	Fin spacing, (mm).	2
Thickness of tube, (mm).	0.15		

### The nanofluid’s production

**Fig. 2:**
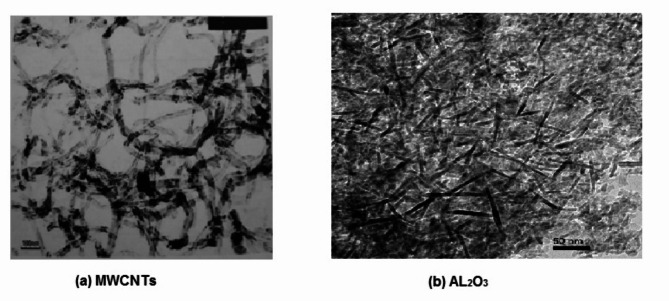
Image of nanoparticles obtained using transmission electron microscopy.

**Fig. 3:**
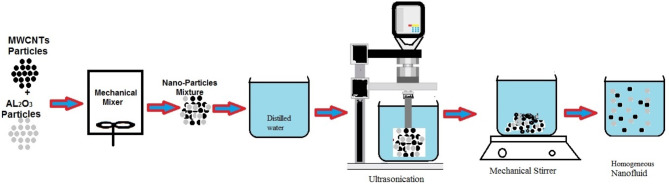
The production of nanofluids is shown diagrammatically.

To increase thermal performance, hybrid nanofluids have been employed recently. There are several applications for MWCNTs and Al_2_O_3_ nanoparticles. Numerous investigations have used both computational and experimental methods to examine MWCNTs, Al_2_O_3_, and single nanofluids. In the present examination, MWCNTs-Al_2_O_3_/water hybrid nanofluids were generated using MWCNTs and Al_2_O_3_. Sigma-Aldrich Chemicals, USA is where Al_2_O_3_ was obtained, and Cheap Tubes Company, USA, was where MWCNTs were obtained. A TEM picture of MWCNTs with Al_2_O_3_ nanoparticles is displayed in Fig. 2. MWCNTs- Al_2_O_3_/water nanofluid with a particle volume concentration of (50:50). Table 2 displays the particle specifications. To develop a hybrid nanofluid, MWCNTs and Al_2_O_3_ nanoparticles were mixed in a mechanical mixer at four distinct volume concentrations: 0.5%, 1.1%, 1.8%, and 2.3%. In Fig. 3, as displayed, To the approximately 10 l of water that fills the tank, the nanoparticle combination is made ahead of time and added under the necessary volumetric concentration. Applying ultrasonication for 1 h to the particles promotes their dissolution throughout the main fluid, hence mitigating the agglomeration process that occurs within the water. After that to guarantee uniform dispersion of the particles within the main fluid, the combination is put on a mechanical stirrer for six hours.

**Table 2:** The MWCNTs physical features.

MWCNTs used specifications		AL_2_O_3_ used specifications	
Outer dia.	20–40 nm	Particle size	≤ 50 nm
Length	10–30 μm	Density	3690 kg/m^3^
Purity	> 90%	Specific heat	880 J/Kg K
Density	2100 kg/m^3^		
Specific heat	790 J/Kg K		

### Evaluation of the nanofluid’s stability

A spectrophotometer (DR 2000; HACH) was used to assess the stability of the water-based (MWCNTs + Al_2_O_3_) nanofluids, and the samples were evaluated once a week. According to this, a linear correlation may be observed between the absorbance and the increase in particles volume concentrations. A maximum of 18% of the 2.3% volumetric content may have been sedimented, based on the minimum relative concentration of 1.1 that was reached during the evaluation period. More colloidal stability was seen 28 days after preparation in a sample with a relative concentration of 1.1 and colloidal stability of 0.25%, or 10% sediment. Prior to being used in automobile radiator tests, the results validated the samples’ high level of stability.

When it comes to heat transfer metrics like specific heat capacity and thermal conductivity, nanofluids are new types of fluids that have better thermophysical properties than basic liquids^27^. Equations below are used to assess the properties of nanofluids.

Utilize the following formulas for each volume fraction to estimate the necessary amount of nanoparticles^28^:

1 $$\:\text{Volume}\:\text{concentration},\:{\Phi\:}=\left[\frac{\:1}{\frac{{\rho\:}_{hy}}{{\rho\:}_{w}}\:\left(\frac{1-w}{w}\right)+1\:}\right]$$

where w stands for the mass ratio and ρ_hy_ of the hybrid particles density. ρ_w_ is the water density.

The Pak and Cho formula^29^ is used to determine the density of nanofluid in the following manner:

2 $$\:{{\uprho\:}}_{\text{h}\text{y}}\:=\frac{{m}_{MWCNT}+{m}_{Al2O3}}{\frac{{m}_{MWCNT}}{{\rho\:}_{MWCNT}}\:+\:\frac{{m}_{Al2O3}}{{\rho\:}_{Al2O3}}}$$

3 $$\:{{\uprho\:}}_{\text{n}\text{f}}=\left(1-\varPhi\:\right){\rho\:}_{w}+\:\varPhi\:\:{\rho\:}_{hy}$$

4 $$\:{\text{C}}_{\text{n}\text{f}}=\frac{\left(1-\varPhi\:\right){\rho\:}_{w}\:{C}_{w}\:+\:{\varPhi\:}_{Al2O3}{\rho\:}_{Al2O3}\:{C}_{Al2O3\:}+\:{\varPhi\:}_{MWCNT}{\rho\:}_{MWCNT}\:{C}_{MWCNT\:}}{{\rho\:}_{nf}}$$

One might use the following formula^30^ to get the absolute viscosity:

5 $$\:\frac{{\mu\:}_{nf}}{{k\mu\:}_{w}}=1+\frac{\varPhi\:}{0.4}\:+6.2\:{\varPhi\:}^{2}$$

The following formula^31^ may be used to determine the thermal conductivity:

6 $$\:\frac{{k}_{nf}}{{k}_{w}}=\:\frac{\frac{{\varPhi\:}_{Al2O3}{k}_{Al2O3}\:+\:{\varPhi\:}_{MWCNT}{k}_{MWCNT}\:}{\varPhi\:}+2{k}_{w}+2\:\left({\varPhi\:}_{Al2O3}{k}_{Al2O3}\:+\:{\varPhi\:}_{MWCNT}{k}_{MWCNT}\right)-2\varPhi\:{k}_{w}}{\frac{{\varPhi\:}_{Al2O3}{k}_{Al2O3}\:+\:{\varPhi\:}_{MWCNT}{k}_{MWCNT}\:}{\varPhi\:}+2{k}_{w}+2\:\left({\varPhi\:}_{Al2O3}{k}_{Al2O3}\:+\:{\varPhi\:}_{MWCNT}{k}_{MWCNT}\right)+\varPhi\:{k}_{w}}$$

## Computations in the experiment

Some considerations must be taken into account during the process of evaluating and analyzing the performance of the radiator used in the constructed device during the operation process, according to previous experimental studies. There must be stability in the flow of both air and operating fluid. The characteristics selected are based on the mean fluid temperature.

Numerous relationships were employed to assess HyNf’s heat transfer behavior. The working fluid side rate of heat transfer can be calculated in the manner described below^32^:

7 $$\:\text{Q}_{\text{hf}}={\text{m}}_{\text{{hf}}}\cdot \text{C}_{\text{{p,hf}}}\,\text{T}_{\text{{hf,i}}}-\text{T}_{\text{{hf,o}}}$$

The temperatures of the working fluid at the inlet and outflow are denoted by T_hf, i_ and T_hf, o_.

Convective heat transfer coefficient of the hybrid nanofluid was calculated using Eq. (7).

8 $$\:\text{h}_{\text{hf}}=\frac{{Q}_{hf}}{NA{(T}_{b}-{T}_{w})\:}$$

where N is the radiator tube count. The peripheral area of the tube is represented by A (m^2^). T_b_ represents the working fluid’s bulk temperature ($\:{T}_{\text{b}}=\frac{{T}_{\text{h}\text{f},\:\text{i}}+{T}_{\text{h}\text{f},\:\:\text{o}}}{2})$. T_w_ is the temperature of the radiator wall ($\:{T}_{\text{w}}=\frac{{T}_{1}+{T}_{2}{+\dots\:+T}_{12}}{12})$.

Equation (9) is utilized for calculating the operational fluid’s non-dimensional Nusselt number.

9 $$\:{Nu}_{hf}=\:\frac{h\:{D}_{h}}{\:{k}_{hf}}$$

D_h_ represents the hydraulic diameter. K_hf_ is HNF thermal conductivity.

The hydraulic diameter of the tube may be found using Eq. (10):

10 $$\:{D}_{h}=\:\frac{4\:{A}_{c}}{\:P}$$

P is the tube perimeter. A_C_ represents the tube’s cross-sectional area.

Equations (11, 12) are used to calculate the nondimensional Reynolds and Prandtl numbers of the working fluid on the tube side.

11 $$\:{Re}_{hf}\:=\:\frac{\varvec{v}\varvec{*}\:{D}_{h}}{{\varvec{\nu\:}}_{\text{h}\text{f}}}$$

12 $$\:{Pr}_{hf}=\:\:\frac{{{\varvec{\mu\:}}_{\text{h}\text{f}}\:C}_{p,\:\:hf\:}}{{\varvec{k}}_{\text{h}\text{f}}}$$

The formula below is utilized to calculate the Darcy friction factor based on the pressure differential in the mercury column:

13 $$\:\text{f}=\frac{{\Delta\:}\text{P}}{\left(\frac{\varvec{N}\varvec{*}{\varvec{L}}_{\varvec{t}\varvec{u}}}{{D}_{h}}\right)\left(\frac{{\varvec{\rho\:}}_{\text{o}\text{f}}\varvec{*}{\varvec{v}}^{2}}{2}\right)}$$

The theoretical Nusselt number for the turbulent flow may be obtained using the Dittus and Boelter correlation^33^.

14 $$Nu_{hf}= 0.023 (Re_{hf})^{0.8} (Pr_{hf})^{0.3}	$$

According to^34^, the overall effectiveness of cross-flow unmixed/unmixed compact heat exchangers is calculated as outlined below:

15 $$\:\epsilon\:=1-\text{exp}\left(\frac{1}{{C}^{*}}\right)\left({NTU}^{0.22}\right)\left[exp\left({-C}^{*}\left({NTU}^{0.78}\right)\right)-1\right]$$

The following definitions apply to $\:{C}^{*}$ and NTU in this equation^35^:

16 $$\:\:\text{N}\text{T}\text{U}=\:\frac{UA}{{C}_{min}}$$

17 $$\:{C}^{*}=\:{C}_{min}/{C}_{max}\:=\:\frac{{\left(\:{m}^{.}{c}_{p}\right)}_{min\:}}{{\left(\:{m}^{.}{c}_{p}\right)}_{max\:}}\:$$

Throughout experimentation, uncertainty is used to identify measurement mistakes. Measurement errors such as systematic and random errors are the cause of these uncertainties. Researchers such as Kline, McClinton, and Moffat^36^ have devised several approaches to understand those errors; one such methodology that they described is RSS (Root Sum Square Method). Assume for the moment that we have a function f that is measured by the three parameters x, y, and z. The uncertainties encountered during the measurement are represented as dx, dy, and dz. Equation (18), obtained by the RSS approach, is obtained.

18 $$\:\delta\:f=\sqrt{{\left(\frac{\delta\:f}{\delta\:x}\delta\:x\right)}^{2}+\:{\left(\frac{\delta\:f}{\delta\:y}\delta\:y\right)}^{2}+{\left(\frac{\delta\:f}{\delta\:z}\delta\:z\right)}^{2}}$$

For the different measurement parameters, Table 3 shows the accuracy and uncertainty of the equipment.

**Table 3:** Uncertainty in experimental results.

Alteration on the parameters		Derived relative uncertainty ranges through experimentation	
Parameter	Accuracy	Parameter	error
Temperature	*±* 0.1^o^C	Q	*±* 5.2%
Flow rate	*±* 0.1 l/min	h	*±* 6.32%
Particles weight	*±* 0.001 g	Re	*±* 4.76%
Manometer	*±* 1 mm	Nu	*±*5.74%
		f	*±* 3.74%

## Results and discussion

**Fig. 4:**
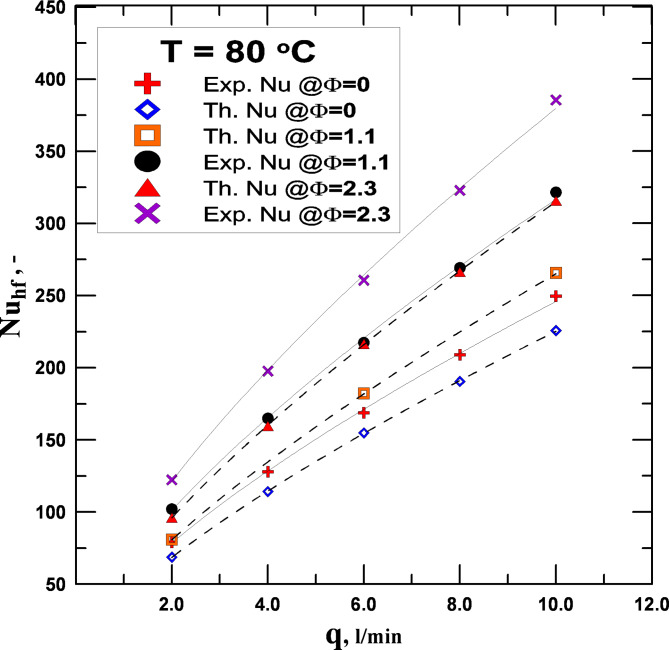
Comparing between exp. and th. Nu no. with variation volumetric flowrate.

The experimental configuration was validated by contrasting the outcomes with correlation-based Nusselt number values that were obtained using nanofluids and distilled water in the radiator. The experimental Nusselt number results were compared with the Nusselt number derived from the Boelter and Dittus relation (Eq. 13) under the conditions of turbulent flow of the working fluids utilized. According to the results, there was a reasonable level of agreement between the data, with an average error rate of 7%. Figure 4 shows a comparison between the theoretical relation for operating fluid with varying concentrations and the Nusselt number calculated empirically. The graphic shows that when the flow rate increases, the Nu also increases, indicating a commensurate increase in the rate at which heat energy is exchanged.

**Fig. 5:**
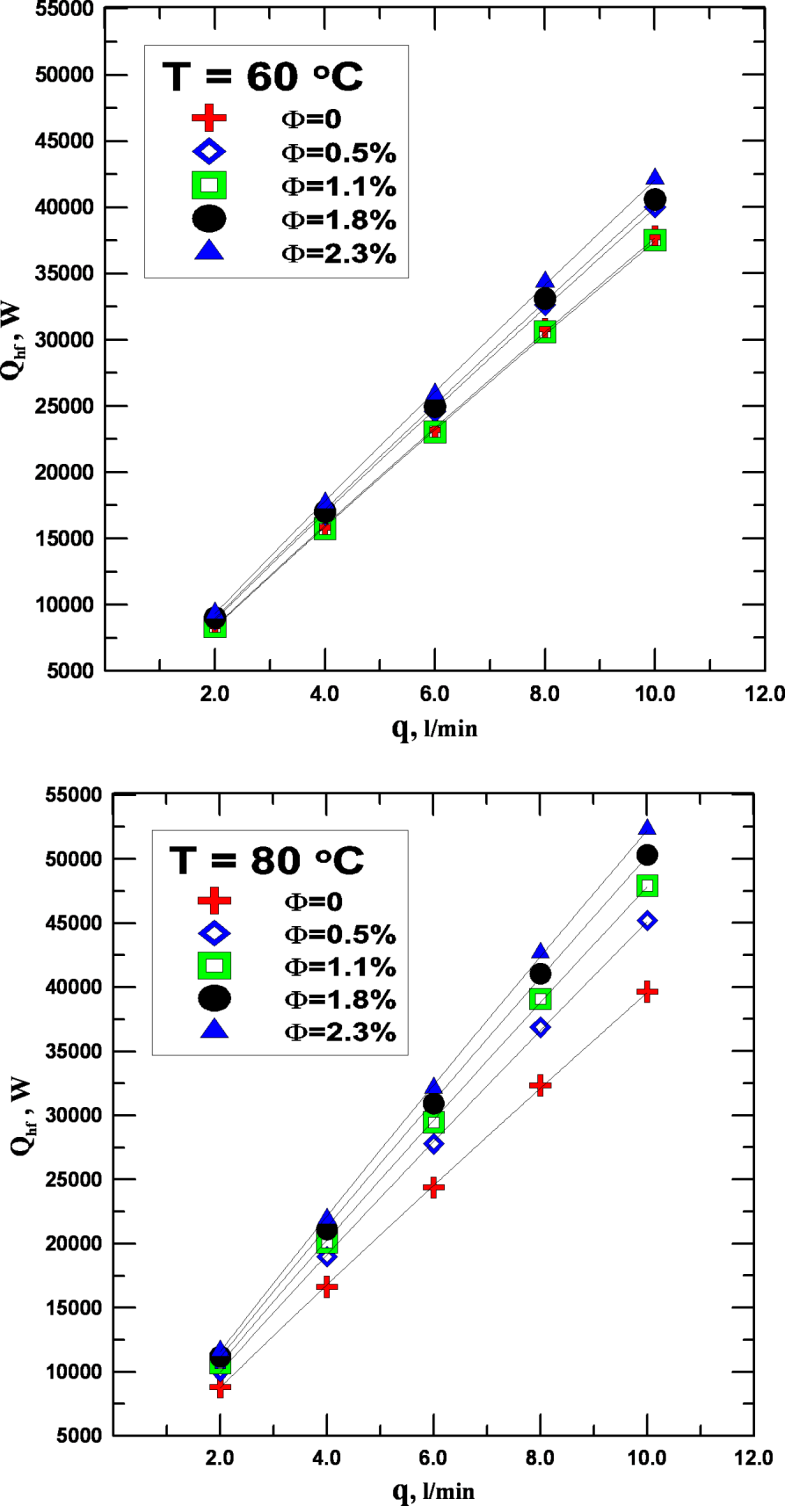
(**a**) Q_hf_ vs. with q for varying values of Φ. (**b**) Q_of_ vs. to q for varying values of Φ.

Figure 5 shows the variation in the flow rate at two different temperatures, 60 °C and 80 °C, together with the rate at which distilled water and hybrid nanofluids transmit heat at different concentrations. The figures demonstrate how the rate of heat transfer increases as the fluid’s flow rate and inlet temperature increase. Two primary causes are responsible for this growth. The first is the employment of nanofluids as an alternative to conventional fluids, which had the most effect on the increase’s worth. As seen in the graphic, the rate of improvement in the heat transfer process improves positively with an increase in the concentration of nanoparticles within the base fluid. Depending on how concentrated the fluid is concerning water, the rate of growth in the heat transfer process might vary from 6 to 33%. Due to the fluid’s larger Brownian motion than that found in conventional fluids, this discovery is seen as strong evidence for the importance of the employment of hybrid nanofluids, which have the effect of enhancing heat transmission. This movement has greater intensity in proportion to the fluid’s concentration. The reader can observe from Fig. 5a, b that the heat transfer process gets better the higher the temperature of the fluid, whichever it is. This makes sense since the value of heat exchange rises as the temperature differential does as well.

**Fig. 6:**
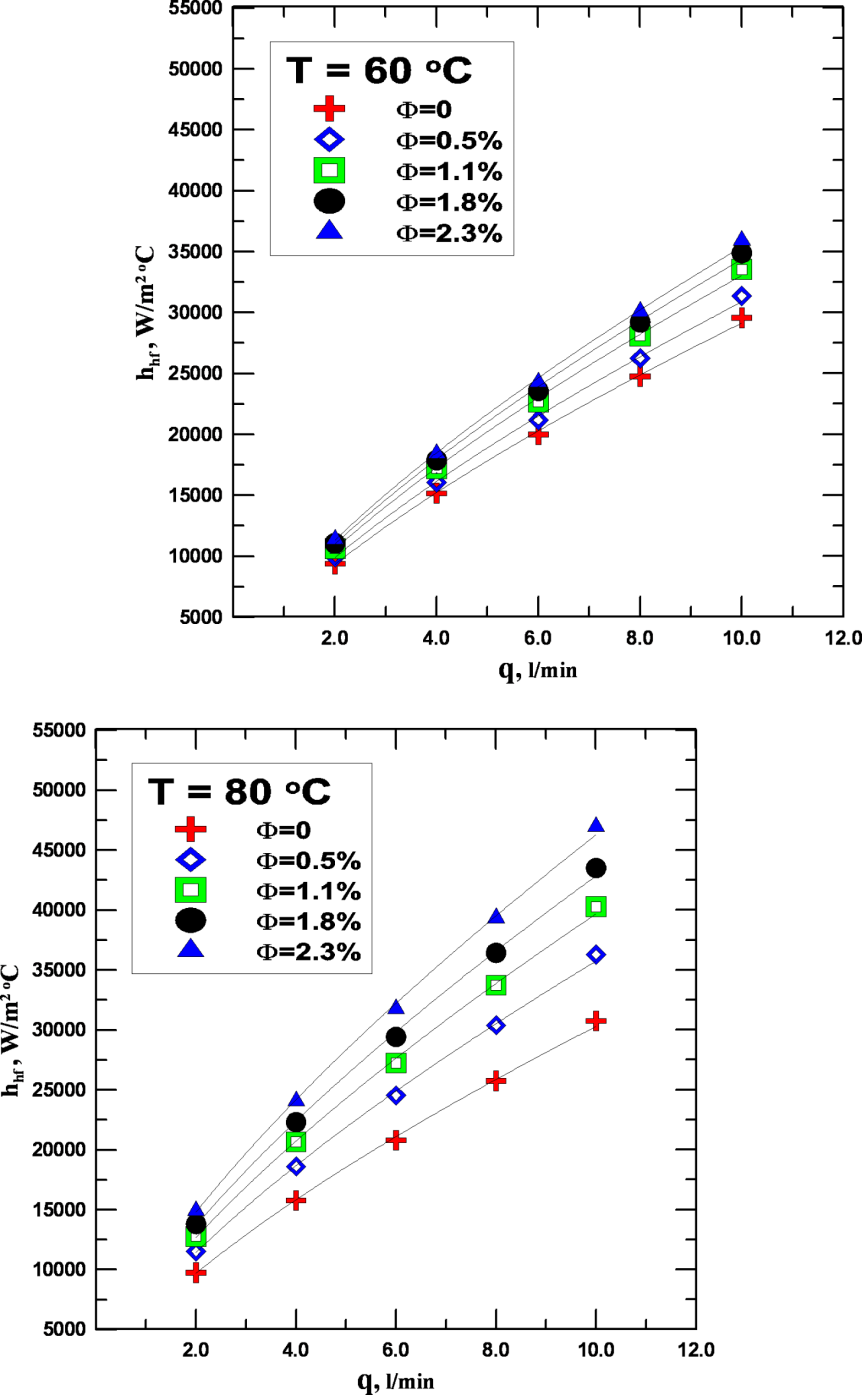
(**a**) h vs. to q for varying values of Φ (T = 60 ^o^C). (**b**) h vs. to q for varying values of Φ (T = 80 ^o^C).

The variation in the heat transfer coefficient (h) for the hot fluid and the fluid flowrate (q) at different temperatures and fluid concentration ratios (Φ) is shown in Fig. 6. Several factors contribute to the figure’s improved heat transfer coefficient values. The values of (h) improve significantly with increasing fluid flow rate from 2 to 10 l/m. This is because the rate of heat transfer improves with increasing fluid flow rate, which in turn raises the increase in (h). Using hybrid nanofluids as the working fluid rather than water is one of the key elements determining the values of (h). When the fluid with a 2.3% concentration was used at the same flow rate and temperature, the rate of rise reached values ranging from 14 to 28.5%. This is because, in comparison with conventional fluids, hybrid nanofluids have better physical characteristics, such as higher thermal conductivity, which aided in accelerating the heat transfer process and enhancing the value of (h). Figure 6a, b make it evident that the temperature rise also significantly accelerated the improvement of the values of (h). Since raising the temperature differential between the hot and cold fluid helps to improve the heat exchange process, the percentage improvement by increasing temperature was between 6 and 14%.

**Fig. 7:**
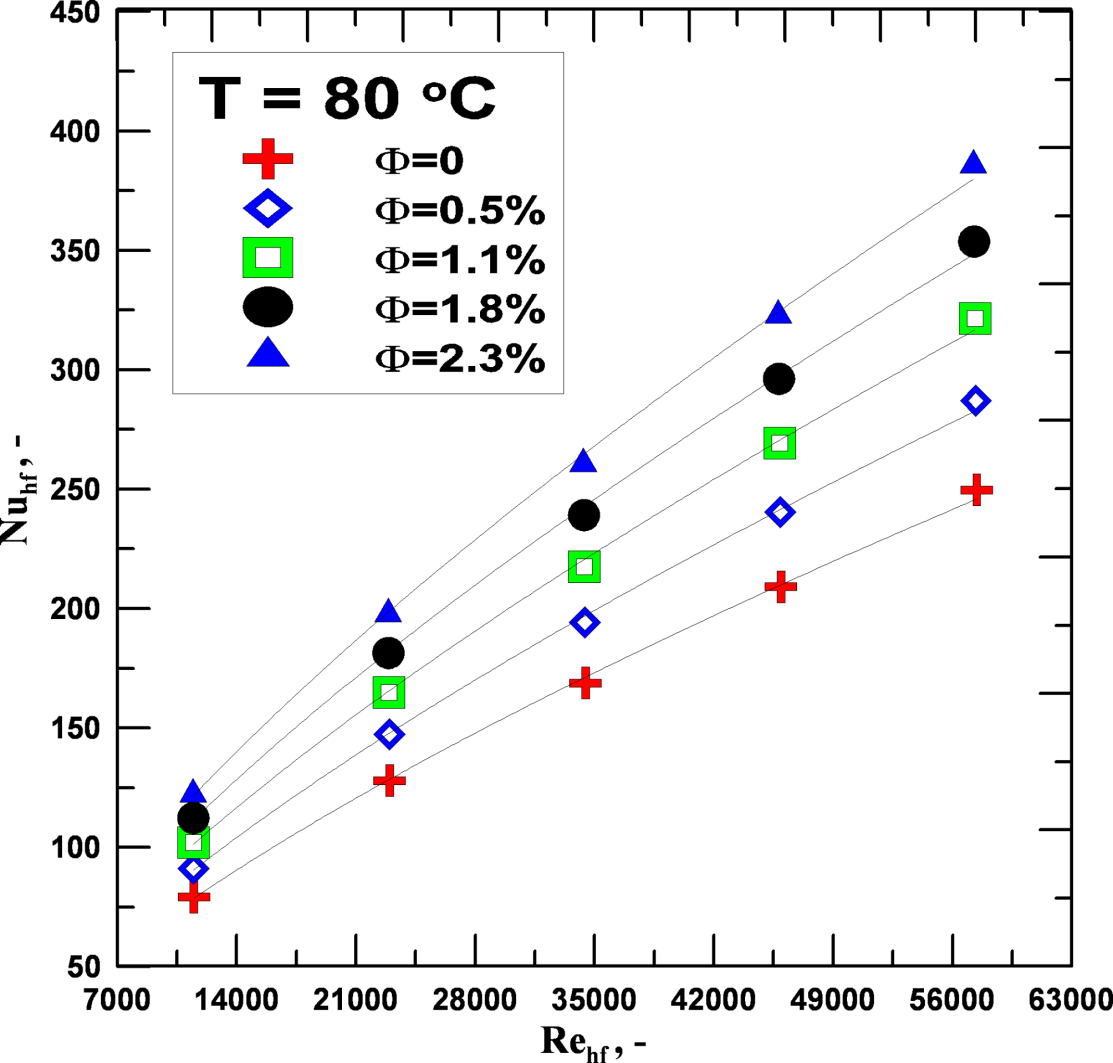
Nu_hf_ vs. to Re_hf_ for varying values of Φ.

The variations in the hot fluid’s Nusselt number (water or hybrid nanofluids) with the Reynolds number and volumetric concentration percentage are displayed in Fig. 7. The Nusselt number increases with increasing fluid concentration and Reynolds number. Nusselt number changes with volume fraction and Reynolds No. have been studied at the same input temperature of 80 °C. At the highest Re, there was the most improvement in the Nusselt number value. When compared to pure water at the same Re, the improvement at this flow rate achieved a minimum value estimated at 6.8% for the volumetric concentration of 0.5%, while the highest rise in the increase reached 23.74% at the volumetric concentration of 2.3%. Utilizing hybrid nanofluids rather than distilled water causes the Nusselt number to increase, which in turn causes the heat transfer coefficient to increase. The improvement in the Nusselt number was mostly caused by the rise in the heat transfer coefficient rather than the fluid’s increased thermal conductivity.

**Fig. 8:**
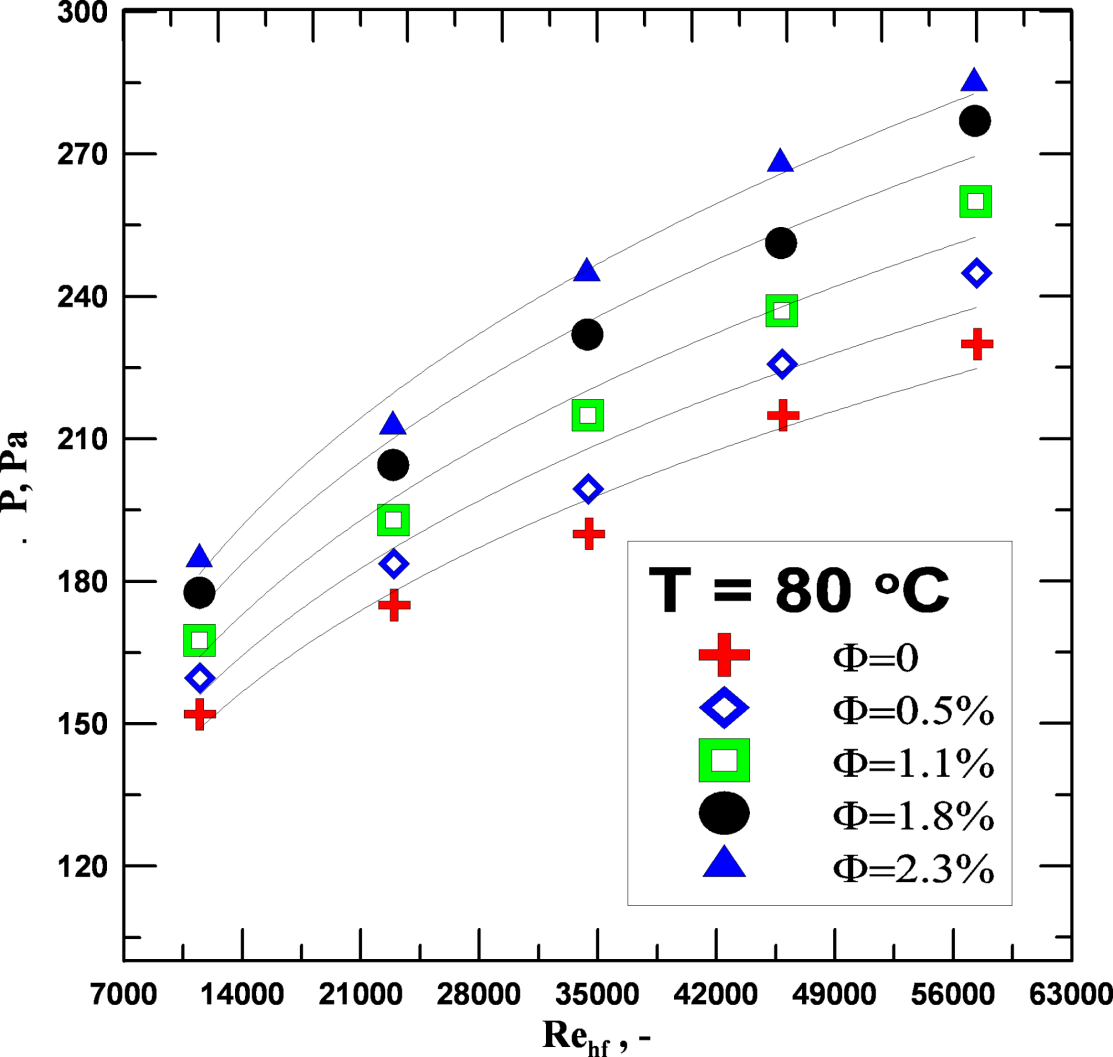
ΔP vs. to Re for varying values of Φ.

Figure 8 shows how pressure drop and Reynolds number are related. As the Re increases, so does the pressure drop. In the regular tube, the pressure drop is dependent on the Re number and volume concentration of the nanoparticles. A higher volume concentration of nanoparticles in the coolant results in a higher fluid density and a higher viscosity in the nanofluids which causes an improvement in the pressure drop. The fluid’s pumping capability is significantly impacted negatively by a drop in pressure. For the same Re number, there is a 6.5–24% increase in the pressure drop rate in the nanofluid than the water depending on the fluid concentration.

**Fig. 9:**
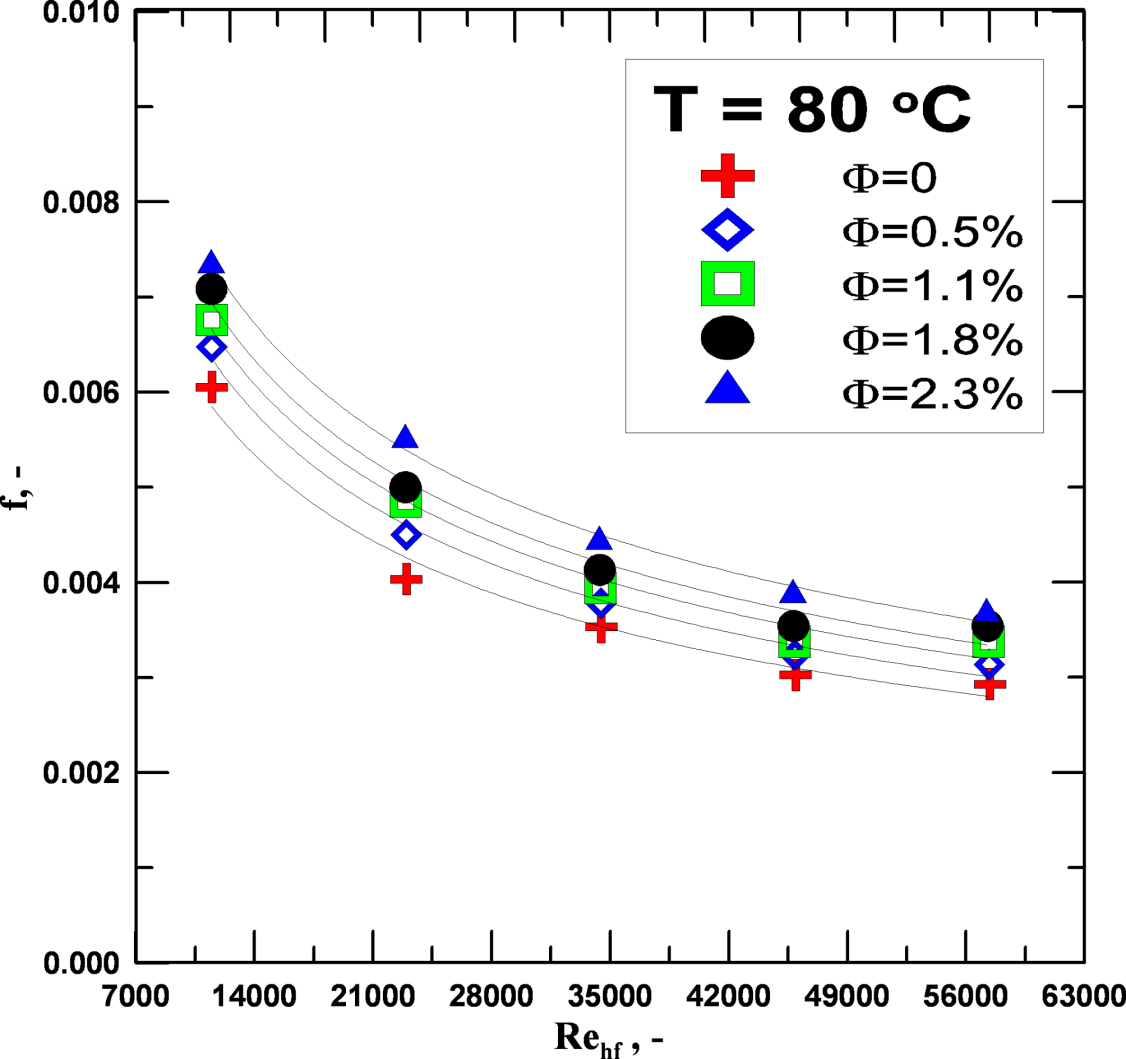
Friction fa. vs. to Re for varying values of Φ.

Figure 9 shows the friction factor (ff) vs. the Re No. for distilled water and nanofluids with varying nanoparticle concentrations and the same initial temperature (T = 80 °C). The findings indicate that, for all nanoparticle concentrations, the ff reduces as the Re No increases. As the ratio of nanoparticle concentration drops, so does the ff. Depending on the rise in the concentration of the nano-hybrid fluid employed, the primary cause of the increase in ff is the increase in pressure drop. In return for a little increase in the fluid pumping force within the radiator, increasing the flow rate is an excellent way to reduce the higher ff. The fluid concentration determines the percentage increase in the ff between 5.7 and 24.3% for nanofluids than distilled water.

**Fig. 10:**
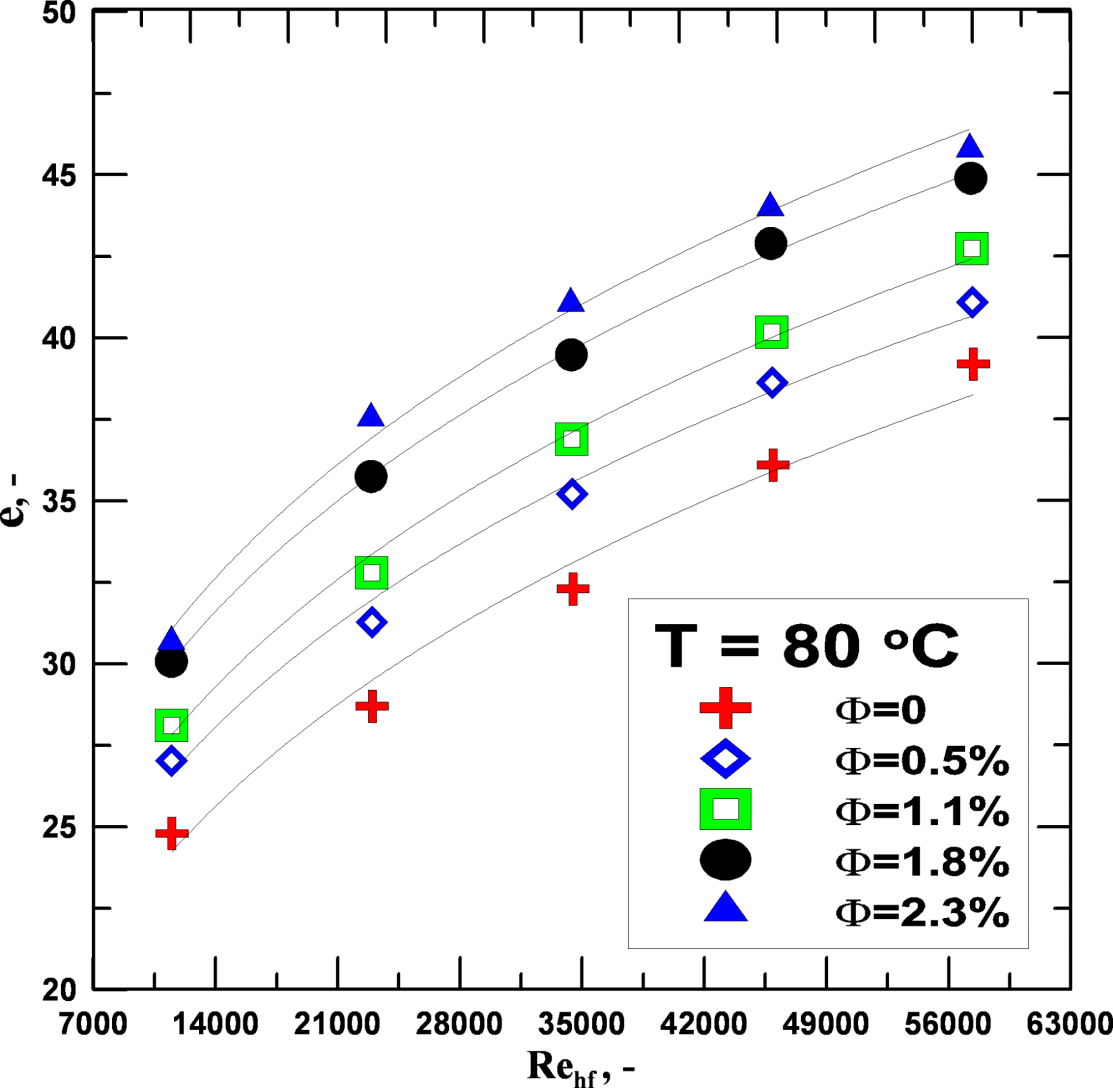
*ε* vs. to Re for varying values of Φ.

Figure 10 investigated the impact of varying the Re No. of the working fluids, which included distilled water and hybrid nanofluids with varying concentrations on the effectiveness (*ε*), at a constant operating temperature of 80 °C. The effectiveness of hybrid nanofluids is superior to that of pure water, as can be shown by analyzing the figure. The larger the concentration of nanofluid in the base fluid, the higher the *ε* value. Also, a significant contribution to enhancing *ε* comes from the Re No. The primary indicator of *ε* for the hybrid nanofluid is an increase in the number of transfer units (NTU). For the hybrid nanofluid with a concentration of 0.5%, the minimum percentage increase in the *ε* value was around 7.4%. When compared to the *ε* values of distilled water under the same conditions, the maximum reached around 22.54% for nanofluids with a concentration of 2.3%.

With the use of performed experiments and research into the variables influencing the enhancement of vehicle radiator performance. To determine the value of the Nusselt number, an experimental correlation (19) was suggested. The proposed correlation is a function of the percentage volume concentration of the hybrid nanofluid (MWCNT–Al_2_O_3_/water) and the mixing ratio (50:50) for each of the nanoparticles used. The correlation is also a function of the Reynolds number with values ranging between 11,000 and 58,000 and 0% ≥ Φ ≥ 2.3%.

19 $${\text{Nu}}={\text{1}}0.00{\text{3 }}{\left( {{\text{Re}}} \right)^{0.{\text{5}}0{\text{5}}}}{({\text{F}})^{0.{\text{367}}}}$$

**Fig. 11:**
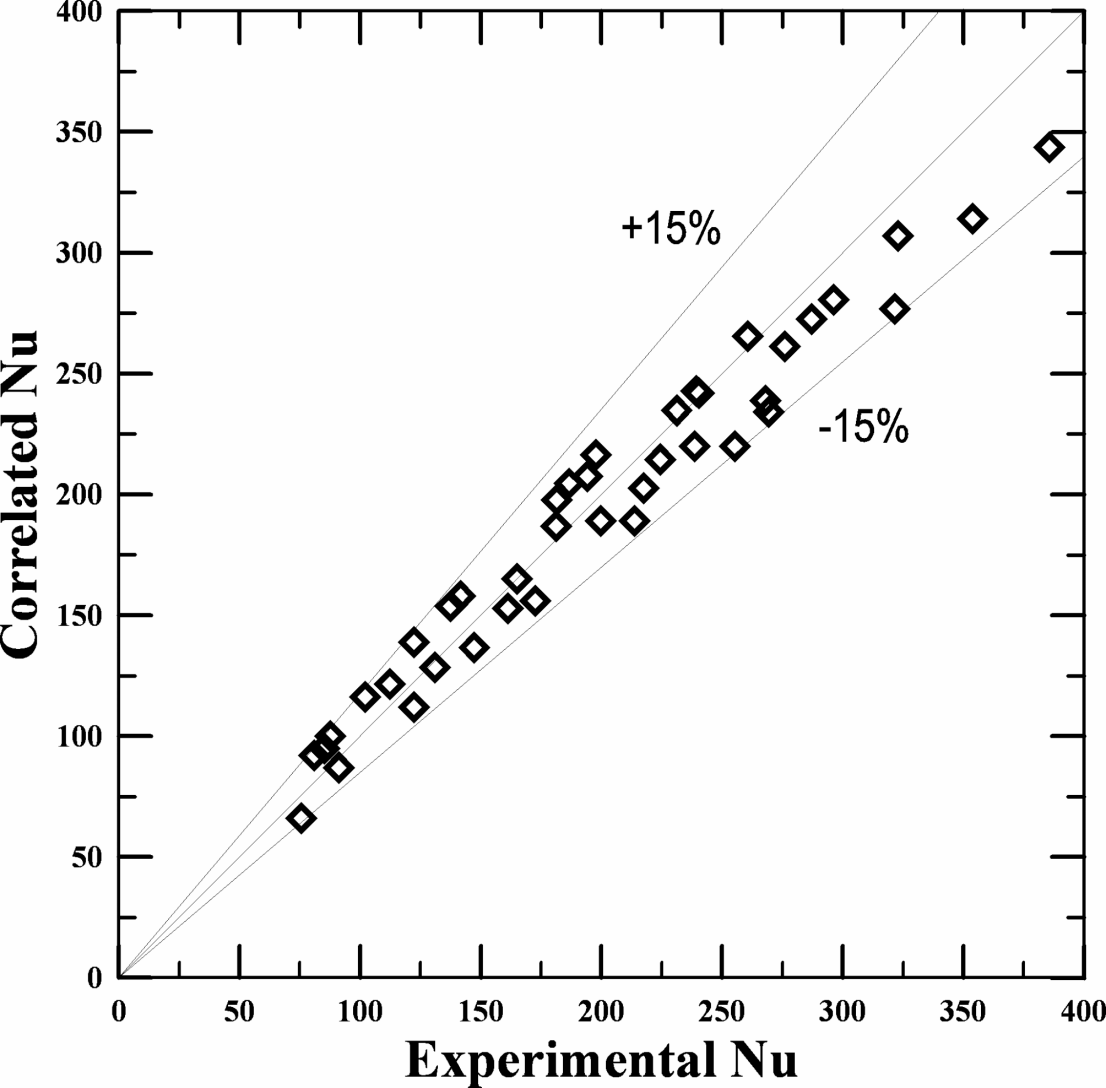
Nu values computed from the proposed correlation; Eq. (18) are compared to the ex. values.

A comparison of the estimated Nu based on the suggested correlation and the empirically determined Nu is shown in Fig. 11. As can be seen from the figure, the greatest margin of error in the values predicted for Nu based on the previously outlined relationship is about ± 15%.

**Fig. 12:**
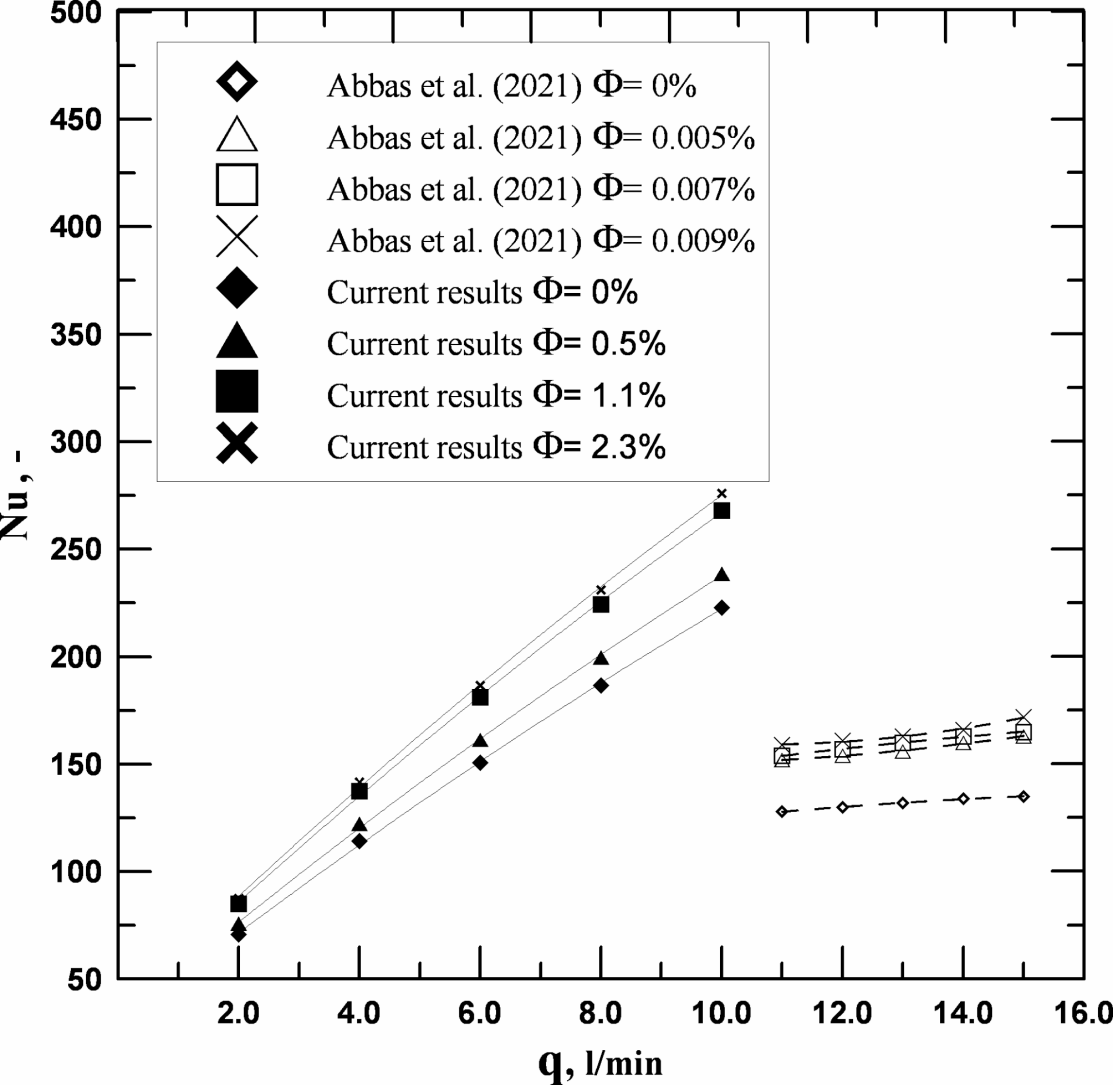
Nu vs. q: A comparison of the current study’s findings with those of Abbas et al. (2021).

It was vital to compare the current research’s findings with those of earlier, related studies to guarantee the validity of the findings. Figure 12 in this study compares the Nusselt number change with the volumetric flow rate with the conclusions of Abbas et al.^14^. The reader may discern a respectable level of concordance between the findings of Abbas et al. and the current study.

## Conclusions

Hybrid nanofluids have become one of the important issues in research and development, especially in the field of heat transfer. These fluids have unique physical properties that distinguish them from traditional fluids. The current research was focused on testing the performance of hybrid nanofluid the MWCNT–Al_2_O_3_/water with different concentrations (0.5, 1.1, 1.8, and 2.3%) to act as a coolant in a vehicle radiator. A vehicle cooling cycle was fabricated to complete the test, consisting of a pump, a radiator of a 2005 Honda Civic, and a cooling fan, the airspeed was fixed (15 m/s). The cycle was equipped with a set of devices to measure temperature, flow rate (2–10 l/min), and pressure loss. After concluding the experiments, the current results were drawn from the study:

- The hybrid nanofluid at all concentrations used in the research is superior to distilled water in heat transfer rate. The heat transfer rate improved by between 6% and 33%.
- There is an increase in the value of the Nusselt number due to the concentration of the fluid used, as it reached 6.8% when using a fluid with a concentration of 0.5%, until it reached 23.7% for a concentration of 2.3%.
- The energy consumption rate of the pump increases due to the increase in both pressure drop and friction factor. The increase in pressure drop rate and friction factor reached 24 and 24.3%, respectively.
- The effectiveness of the radiator increased when using the fluid under study compared to the conventional fluid by a percentage ranging between 7.4 and 22.54%.
- An experimental correlation is proposed to calculate the value of the Nusselt number as a function of the Reynolds number and (Φ).
- By comparing the current work with previous studies, there is a good agreement.

## Data Availability

The datasets used and/or analyzed during the current study are available from the corresponding author on reasonable request.

## Abbreviations

A: Surface area, m^2^
C: Specific heat, J/Kg K
D: Diameter, m
ff: Fraction factor, –
h: Convection heat transfer coeff., W/m^2^ K
k: Thermal conductivity, W/m K
U: Overall heat transfer coefficient, W/m^2^ K
v: Velocity, m/s
m: mass flow rate, Kg/s
q: Volume flow rate, l/min
Q: Rate of heat dissipated, W
Re: Reynolds number (ρvd/μ)
Nu: Nusselt number (hd/k)
T: Temperature, K

## Greek symbols

µ: Absolute viscosity, kg/(m·s)
Φ: Volume concentration, –
ρ: Density, kg/m^3^
ε: Effectivness

## Subscripts

b: Base
hf: Hybrid-nanofluid hot fluid
c: Cold
h: Hot, hydraulic
i: Inner, inlet
o: Outer outlet
p: Particles
t: Total
w: Water
